# Investigating dose homogeneity in radiotherapy of oral cancers in the presence of a dental implant system: an in vitro phantom study

**DOI:** 10.1186/s40729-021-00372-5

**Published:** 2021-09-06

**Authors:** Goli Khaleghi, Hoda Mahdavi, Seied Rabi Mahdavi, Benyamin Khajetash, Alireza Nikoofar, Mohammad Hosntalab, Mahdi Sadeghi, Reza Reiazi

**Affiliations:** 1 Medical Radiation Engineering Department, Science and Research Branch, Islamic Azad University, Daneshgah Blvd., Simon Bolivar Blvd., P.O. Box: 14515-775, Tehran, Iran; 2 Radiation Biology Research Center, Iran University of Medical Sciences, Shahid Hemmat Highway, P.O. Box: 14665-354, Tehran, Iran; 3 Radiation Oncology Department, Iran University of Medical Sciences, Firoozgar hospital, Beh-Afarin St., Karimkhane-Zand Blvd., P.O. Box: 1593747811, Tehran, Iran; 4 Medical Physics Department, School of Medicine, Iran University of Medical Sciences, Shahid Hemmat Highway, P.O. Box: 14155-6183, Tehran, Iran; 5 Princess Margaret Cancer Center, University Health Network, 101 College Street, P.O. Box: M5G 1L7, Ontario Toronto, Canada

**Keywords:** Dental implant, Phantom, Film dosimetry, Density correction

## Abstract

**Background:**

Materials with high atomic numbers are part of the composition of dental implant systems. In radiotherapy of oral cavity cancers, an implant can cause dose perturbations that affect target definition, dose calculation, and dose distribution. In consequence, this may result in poor tumor control and higher complications. In this study, we evaluated dose homogeneity when a dental implant replaced a normal tooth. We also aimed to evaluate the concordance of dose calculations with dose measurements.

**Materials and methods:**

In this study, 2 sets of planning CT scans of a phantom with a normal tooth and the same phantom with the tooth replaced by a Z1 TBR dental implant system were used. The implant system was composed of a porcelain-fused-to-metal crown and titanium with a zirconium collar. Three radiotherapy plans were designed when the density of the implant material was corrected to match their elements, or when all were set to the density of water, or when using the default density conversion. Gafchromic EBT-3 films at the level of isocenter and crowns were used for measurements.

**Results:**

At the level of crowns, upstream and downstream dose calculations were reduced when metal kernels were applied (M-plan). Moreover, relatively measured dose distribution patterns were most similar to M-plan. At this level, relative to the non-implanted phantom, mean doses values were higher with the implant (215.93 vs. 192.25), also, new high-dose areas appeared around a low-dose streak forward to the implant (119% vs. 95%).

**Conclusions:**

Implants can cause a high dose to the oral cavity in radiotherapy because of extra scattered radiation. Knowledge of the implant dimensions and defining their material enhances the accuracy of calculations.

## Background

Radiotherapy brings the opportunity to control tumoral remnants at the post-surgical bed of oral cancers, and also, it is sometimes the only choice for definitive treatment of unresectable cancers of the oral cavity. It is estimated that 1–4% of radiation therapy patients have a prosthetic device that may interfere with dose delivery, plan design, and verification [1]. An increasing number of patients have modern dental restorations that are composed of biocompatible metals such as titanium. Zirconium is increasingly considered in new dental implants for its known properties of minimizing bacterial colonization and reducing plaque formation. It has induced less inflammation and hypersensitivity reactions and also has shown high stability [2, 3]. Mass densities of P-F-M crowns of implant systems and zirconium are higher than titanium, and therefore, their artifacts in imaging, interaction with higher energy photons in radiotherapy, and the amount of backscattered dose can be considerable [3–5]. These materials cause beam hardening and streaking artifacts in CT images and therefore conceal volumetric information and boundaries of radiotherapy targets. Additionally, Hounsfield unit (HU)-to-electron density conversion curves that are incorporated in Treatment Planning Systems (TPS) do not produce reliable outputs in high Z materials [6–8], so they misrepresent density information and alter global dose calculations. Moreover, attenuated beam penetrating the high Z material and lateral scatter cause dose inhomogeneities in Planning Target Volume (PTV) in head and neck cancer radiotherapy that can affect Tumor Control Probability (TCP) [9]. Imaging quality may be improved by metal artifact reduction (MAR) algorithms and extending the CT scale; however, these solutions have not always shown efficacy [10].

Modern collapsed cone convolution/superposition algorithms (CCCS) used for photon dose calculations pose the advantage of lateral scatter calculations in commercial Treatment Planning Systems (TPS) have weaknesses in calculations at metal interfaces which their clinical significance is undetermined [1]. Density correction metal kernel solutions are algorithms that can account for the attenuation of devices and are incorporated in a number of TPSs. According to Physicists in Medicine’s Task Group 63 recommendations ignoring the presence of the prosthesis by setting their density to water, or modifying beam orientations are suggested approaches that have been used in radiotherapy plans to reduce inevitable calculation errors in presence of metal devices in the hip [6, 7]. Meanwhile, because there are no universal practical recommendations for radiotherapy planning when beams penetrate a dental implant, and these devices are not removed before radiotherapy, in this in vitro study a simplified oral cavity phantom was used for point-to-point POI and ROI dose value and distribution comparison of calculated and measured film dosimetry when an implant replaced a normal tooth. Radiochromic films are a prime candidate for dosimetry when demanding high spatial resolution and conditions of charged particle disequilibrium [11]. Therefore, volumes of compositions of the implant system were contoured and calculations were compared to film dosimetry measurements. Relative dose homogeneity at a 2-dimensional level of crowns that represented the soft tissue of the oral cavity was also assessed. We aimed to answer questions of, (1) How does the titanium, zirconium, P-F-M dental implant system affect dose distribution upstream and downstream to the implant in a simplified field, and (2) Do specifying material for contoured components of an implant and applying metal kernels improve dose calculations?

## Materials and methods

### Phantom

**Fig. 1:**
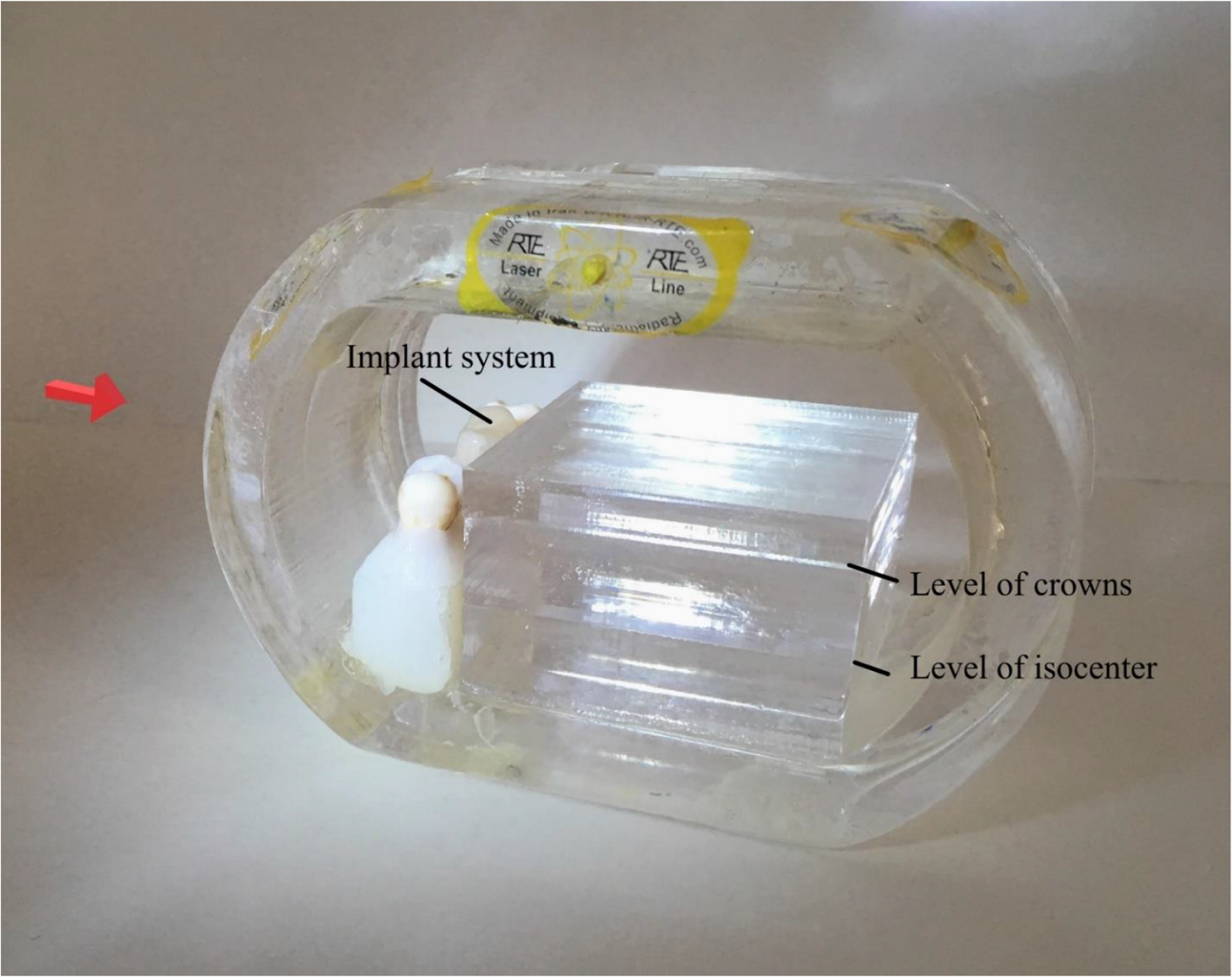
Oral cavity phantom made of Perspex, Teflon, and teeth. The placement of EBT-3 films at two levels is shown. The red arrow shows the direction of the radiotherapy beam

A modeled coronal section of an oral cavity phantom made of Perspex (the equivalent of tissue), Teflon (the equivalent of mandible), and teeth were used for our study as described by Jabbari et. al [12]. Roots of three molar teeth were inserted inside the screwed modeled jaw. The teeth were then fixed and surrounded by soft tissue-equivalent bolus pellets (Orfit, Wijnegem, Belgium) (Fig. 1). The middle tooth was later replaced by a Z1410 dental implant that was composted of a titanium core and a zirconium collar, and abutment (TBR Implants Group, Toulouse, France). The implant system carries a porcelain-fused-to-metal (PFM) crown that includes an alloy of mainly CO and Cr. Two Perspex layers of 4.0 × 4.0 ×1.0 cm and one layer of 4.0 × 4.0 × 0.5 cm represented the soft tissue of the oral cavity including the tongue. Location of Gafchromic EBT-3 films (Ashland ISP Advanced Materials, NJ, USA) was set for two areas at different depths in the horizontal plane between Perspex layers. One film was placed at the level of the roots of the teeth that was also the level of isocenter and the titanium screw where the isocenter was set, and the other one was placed 1 cm above the isocenter, at the uppermost level of crowns (UL) with the same dimension as Perspex layers. A third film (3×4 cm**)** was placed at a slot on the concave side of the phantom.

### Equipment

The dose was delivered using a compact linear accelerator (Elekta Ltd., Stockholm, Sweden) equipped with 6 MV photons. CT images were acquired on a syngo CT VC40 (Siemens, Shanghai Medical Equipment Ltd.) with a slice thickness of 1 mm and an in-plane resolution of 512×512 pixels. The dose calculation was accomplished by CCCS algorithm version 3.5 implemented in RayPlan version 7.0.0.19 (RaySearch Laboratories, Stockholm, Sweden) treatment planning system (TPS) that uses energy corrected effective density water to calculate dose-to-different-density-water [13]. The dose grid size was set to 1 mm. The density of the region of interest (ROI) can be manually set to a specific mass density ranging from a value of 10 ^-3^ to 22.57 g/cm^3^.

### Calibration of dosimetry films

Films were cut to 2 ×2 cm dimensions and exposed by doses of 0.5, 1, 1.5, 2, 3, and 4 Gy for calibration. Monitor unit (MU) values for all exposures were calculated. A 30×30×30 cm^3^ phantom was created in TPS and then MUs of different dose values were calculated at a depth of 5 cm of the central axis and field size of 10×10 cm^2^. For calibration, a 30×30×30 cm^3^ Perspex phantom was used with the same setup. For calibration validity, a 0.6 cc Farmer-type ionization chamber (PTW model TM 30013) was used at a 5-cm depth. Exposures were repeated 3 times for reproducibility. Mean doses to the central area of 0.014 cm^2^ of the films were read. All calibration films were scanned by ScanMaker 9800XL (Microtech CO., Hsinchu, Taiwan) after 48 h. The central area of 0.014 cm^2^ was selected for each film in ImageJ software (v1.52a, USA), the mean value of optical density *(OD*_*n*_*)* in the area was read and it was used to calculate *NetOD = LOG*_*10*_ *(OD*_*n*_ *– OD*_*0*_*)* where the *OD*_*0*_ is the optical density of non-irradiated control film. Dose values were calculated by using a dose-optical density calibration histogram.

### Planning

CT planning images of non-implanted and implanted phantoms were taken with a 1-mm thickness in a reclining position in which the row of teeth was along Z axis. Margins of films and tooth implants were contoured. The crown, abutment, and screw were separately contoured based on their dimensions https://www.medicalexpo.com/prod/tbr-implants-group/product-74246-724926.html. The 6 MV lateral beam, perpendicular to the row of teeth, was planned with SSD=95.1 and field size=10×10 cm^2^. The plan was run 3 times when densities of the material were not set for the implant components, when they were set to water 1 g/cm^3^, and when the densities of the components were set manually in TPS software. Hence, mass densities of the crown were set to 8.8 g/cm^3^, zirconium 6.51 g/cm^3^, and titanium 4.54 g/cm^3^. The rationale was the CT-to-electron density conversion of the planning system may not be reliable when high Z material is used [6]. The PTV of this plan was the representative tongue soft tissue and floor of the mouth.

### Dosimetry

The films were put inside the phantom for radiation. The measurement was repeated 3 times for both setups of the phantom, i.e., with and without the implanted tooth. Dose profiles and mean doses of EBT-3 films were calculated using an in-house code in MATLAB R2019a (Math Works, MA, USA). Homogeneity indices (HI) were calculated using *D*_*2*_ *-D*_*98*_ */D*_*p*_*×100* in which D_p_ is the prescribed dose, D_2_ is the value of dose in 2% volume, and D_98_ is the value of dose in 98% volume. The lower value indicates a more homogenous dose distribution within the volume.

## Results

The HU value of the implant system saturated at 3071 uniformly. Consequently, visual discrimination of implant components was unattainable and we had to rely on the manufacturer’s dimensions from the catalog. After planning, a single 6 MV photon field when 200 cGy was prescribed to the isocenter. Calculations based on density conversion curves for normal teeth were not significantly affecting the MU calculations, as it was observed that the TPS calculated MU of the machine 238 for the non-implanted phantom, identical to the MU when the implant was set to water (W-plan). Noteworthy, M-plan possessed the highest MU that is explained by the beam attenuation effect of the implant. These values were 241 for the implanted phantom when implant material was not manually set or the default plan (D-plan), and 258 for implanted phantom when implant materials were set manually (M-plan). The relative dose uncertainties were calculated ±0.3% in measured and calculated dose, and additionally, the gray level value and location of some points (penumbra and center axis of the beam) in UL and isocenter area were estimated. The dose value of points was calculated by using a dose calibration plot in the penumbra and center axis of each area and compared with TPS as described in [14]. By using this method, the gamma index was equal to 1.8 at the level of isocenter and 2.1 at the level of crowns which both acceptably show the validity of measurement and calculated dose.

**Fig. 2:**
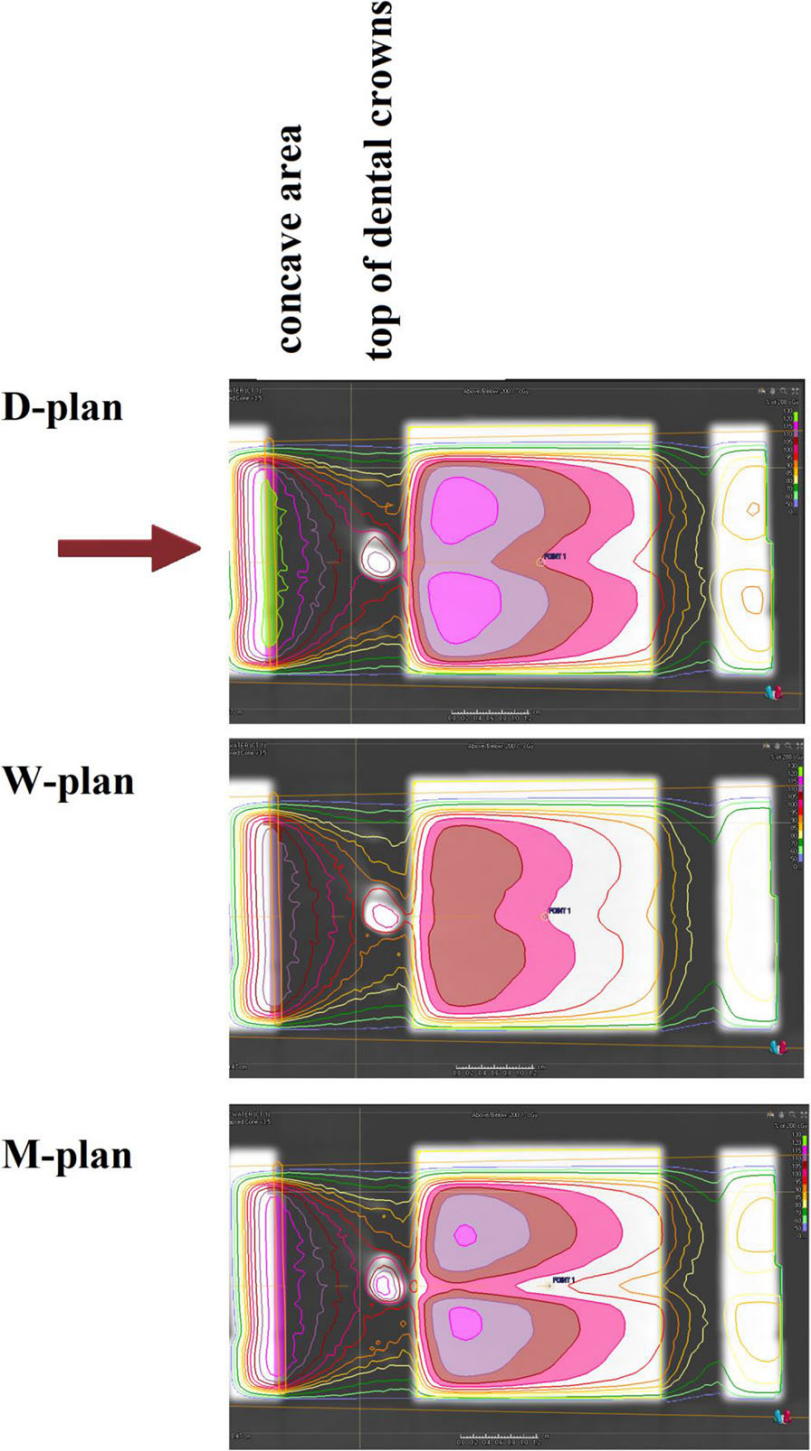
Comparing dose distributions normalized to isocenter between 3 different settings for implant material at the level of crowns. The red arrow shows the direction of the photon beam. Areas with a solid color show isodose levels above 100%. Magenta 100%, rosewood 105%, lavender 110%, and fuchsia 115%

**Fig. 3:**
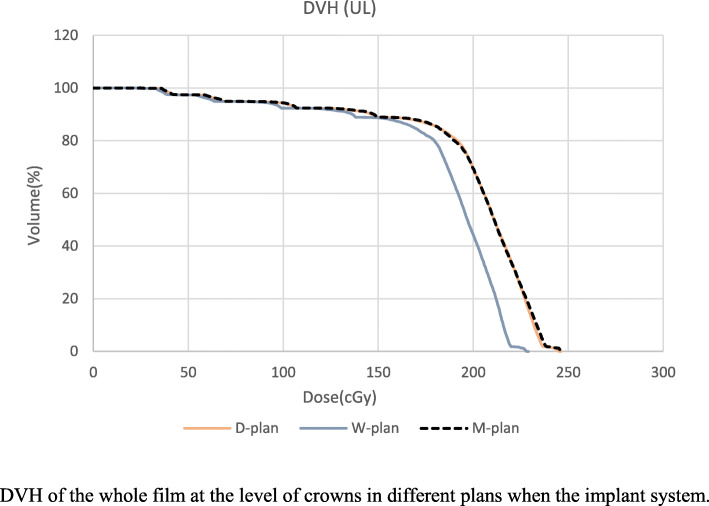
Comparing DVH of the whole film at the level of crowns in different plans when the implant system

**Fig. 4:**
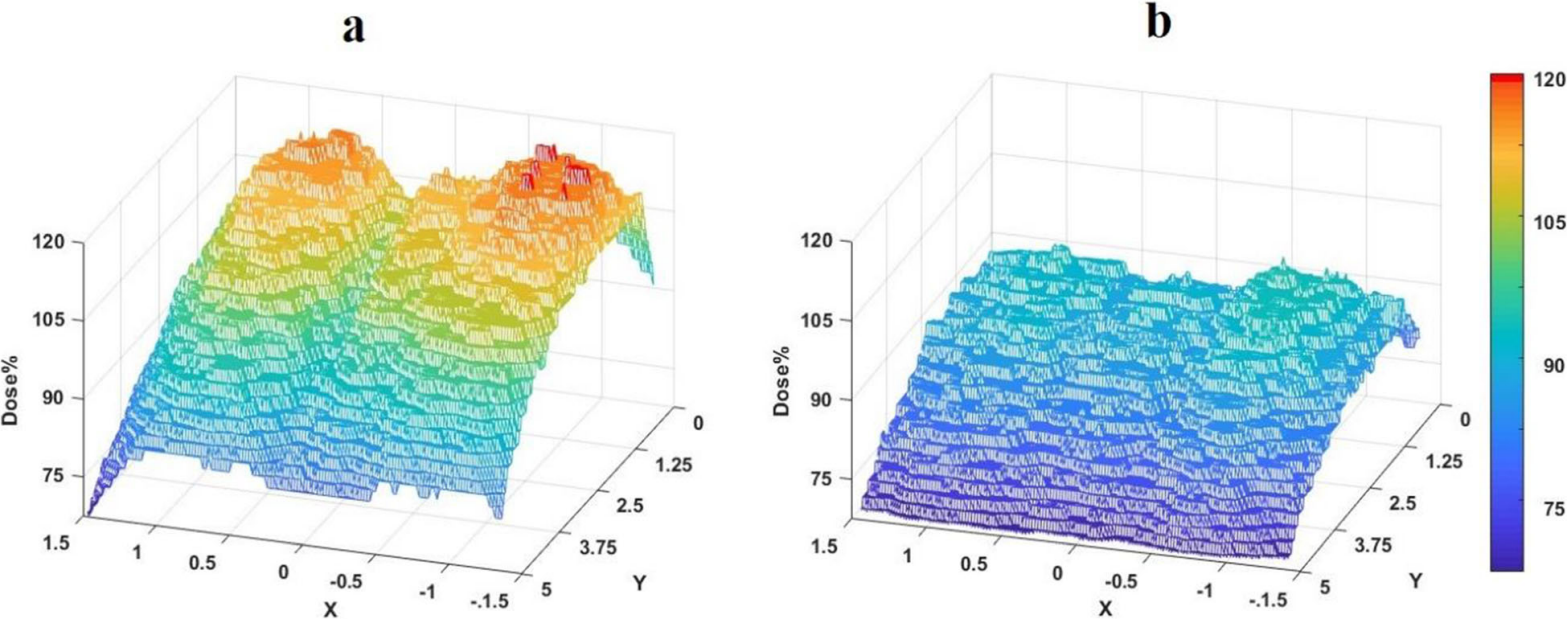
EBT-3 relative dose profiles of the area of 3cm × 5 cm within the field at the level of crowns **a** with and **b** without dental implant

D_1_ (Max dose to 1% of the volume of the film) at the UL was 110.5% for W-plan, 119% for M-plan, and 122% for D-plan. The maximum calculated dose in the M-plan in the whole phantom was 123.5%. Average calculated dose to all films were higher in the implanted phantom in M-plan compared to the non-implanted phantom, particularly, this was 215.93 vs 192.25 at the level of crowns. In this plane areas with 115-119% maximum relative dose emerged at both sides of a ‘shadow’ streak with the implanted phantom (Fig. 2). Dose distribution at the level of crowns was more homogeneous (HI= 91.06%) for W-plan compared to D-plan and M-plan that HI were 97.96 and 98.66 respectively. Dose Volume Histograms (DVH)s of the film at the UL are shown in Fig. 3 which indicates that difference between histograms of D-plan and M-plan is minimal. Measured film dosimetry profiles at the UL in 3×5 cm areas and mean and Standard Deviation of doses calculated by MATLAB software are shown in Fig. 4. Table 1 shows calculated and measured doses of selected points of interest in 2 areas as examples.

**Table 1:** Absolute and relative (percentage) calculated and measured dose by film dosimetry for point U

**U**	**Calculated cGy (%)**		
	Implanted		Non-implanted
	M-plan	198 **(99%)**	-
	D-plan	212 (106%)	197 **(98.7%)**
	W-plan	201 (100.5%)	-
**Isocenter**		200	200
**U**	**Measured cGy (%)**		
	Implanted^*^		Non-implanted
		213 **(98.6%)**	192 **(98.4%)**
**Isocenter**		216	195

*Monitor Unit was set from the default plan which no material was predefined

**Fig. 5:**
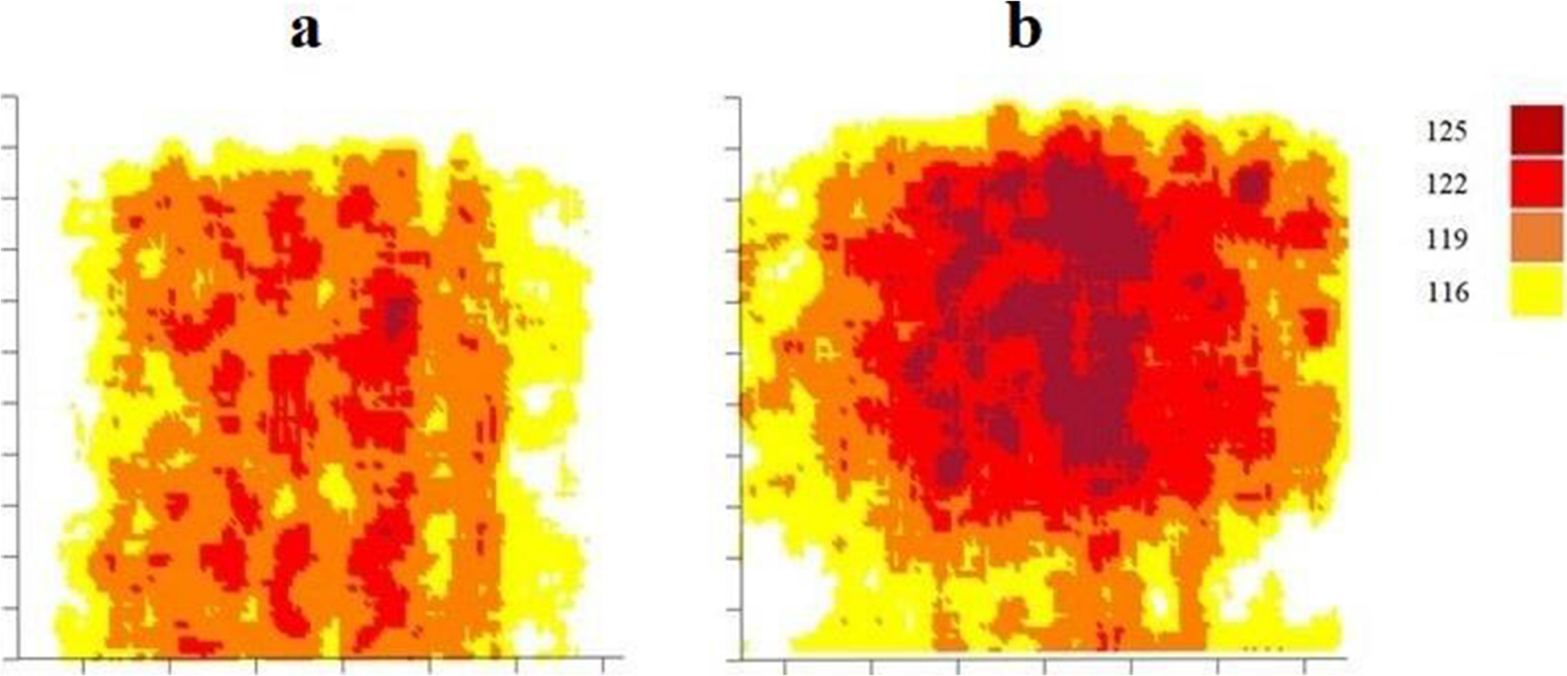
EBT-3 Measured dose distribution at the concave area that represents the buccal surface. The legend shows a dose percentage relative to dose at isocenter **a** without and **b** with an implant. The Y-axis is along the concaveness and the X-axis is along the row of teeth

The concave area showed highest backscatter dose with D-plan (D_1-2_ = 125%), the second was M-plan (D_1-2_= 120%), and the least was W-plan (D_1-2_=114%). D_1-2_ for the non-implanted phantom was 115% at the concave area. As illustrated in Fig. 5, Dosimetry showed that an area of 0.81 cm^2^ received as high as 125-127% on the concave area when the implant was placed that was roughly 3-5% higher than max dose to the non-implanted phantom. Measured dose by EBT-3 films in implanted phantom was validated by calculated dose in implanted phantom when set to default setting in TPS. In this study there was 0.3-0.4% relative dose discrepancy between dosimetry and Rayplan calculations with density corrections for hot areas and the off-center POI in comparison to non-implanted phantom with three repeats of dosimetry. This level deemed acceptable with respect to statistical and systematic uncertainties of Gafchromic film dosimetry that is in order of 0.45% for relative dosimetry provided it is repeated five times [11].

## Discussion

The main findings of point and profile dosimetry of the representative oral soft tissue at the level of crowns were applying metal compositions (M-plan) to the planning system reduced hot areas of PTV and made the plan more resembling film dosimetry compared to (D-plan) that is when dose was calculated with HU-to-electron density conversion curves. In this regard, despite similar DVHs of D and M-plan, relative selected point dose (U) of the planar level of crowns (UL) in ‘implant shadow’ was lower in both M-plan and dosimetry compared to D-plan and W-plan. All these findings show that when inputs of metal kernels are not available, erroneous calculations mislead plan verification.

The results further showed that the backscattered electrons to the concave buccal surface caused an increase in dose to 125-127% (3% higher than baseline) measured with film dosimetry that was close to M-plan calculations. This was predictable as high-density materials induce backscatter electrons. backscatter dose calculations are not correctly predicted in modern CCCS TPSs [10]. However, simplified CCCS are supposed to underestimate the backscatter dose enhancement at proximal interface of an implant but overestimate the dose directly downstream to it [15, 16]. Interestingly, the backscatter dose was the highest in D-plan when compared to M-plan and dosimetry results. This means that the direction of scatter may not have been predicted correctly without metal kernels. Another finding was, relative dose to U was higher both in M-plan (0.3% higher) and measured by film dosimetry (0.2% higher), than the non-implanted phantom. This result shows that the implant had made a global dose increase because of extra scatter even in the shadow area and suggest that PTV near dental implants may unexpectedly receive higher mean dose. Also, areas lateral to the shadow streak showed overdose in M-plan, and as measured by film dosimetry. According to a Tumor Control Probability (TCP) prediction curves, small areas of overdose in the tumor volume does not improve TCP so has no added benefit. Instead, increased scatter dose can adversely increase dose to normal structures such as mucosa of oral cavity, and induce higher mucositis, or increase dose to parotid glands resulting in more frequent xerostomia [17]. However, very small volumes of under-dose can have an enormous effect on TCP [18]. This should be taken into account as non-homogenous dose distribution can lead to underdosed areas when dose levels are adjusted.

Moreover, in our study HU of planning CT saturated at 3071 in all components of implant system and its interfaces, and therefore, the contouring of components of the implant was not feasible before aided by catalogue of the manufacturer. Artifacts can inflate the size of the device and overestimate beam attenuation [10]. This is important as implant geometry can be concealed particularly in devices with different components. Several solutions for planning with presence of a metal device [19], such as metal artifact reduction (MAR) for planning CT scans that improve definitions of device boundaries all have had variable success rates for reducing systematic errors and uncertainties [20, 21]. This study did not use MAR but as we said we manually corrected contour geometry of components. Our results were similar to studies that have shown that accuracy of dose calculation improves when using material-specific energy kernels for applying depth-hardened photon beam in CCCS algorithms [1, 6, 22, 23]. The shape and distribution of energy deposition depends on the composition of interaction medium and the surrounding materials. Calculation algorithms that take into account for lateral heterogeneities, have acceptable accuracy for calculating inhomogeneous media [6, 24], nevertheless, in Rayplan CCCS calculations the energy deposition kernels are based on photon interactions and scatter in water, and these water-based kernels are scaled in dimension based on the local density defined by HU values of the neighboring voxels. Therefore, a water-based kernel does not correctly describe the shape of the true energy deposition in heterogenous media [24]. The difference between dose to water calculations and more accurate calculations that apply dose to medium with radiation transport in the medium (i. e. Monto Carlo simulation) can be largely depending on the mean ionization number of the consisting materials [13]. Default density scaling of CCCS water kernels may be inadequate, and a worse approximation encounters when the difference between its effective atomic number and water is larger [25]. Zirconium containing implants can induce more pronounced streaking artifacts when compared to pure titanium in CT images [3]. Different algorithms of TPSs including Rayplan have shown agreement with radiochromic film measurements in inhomogeneous low densities in a phantom of thorax [26]. For media including higher densities such as those with metal implants which the density and physical dimensions of the implant are used, CCCS methods could accurately calculate dose attenuation. This has also been investigated in Pinnacle CCCS versus Monaco Monte Carlo algorithms in series of head and neck CT set of real patients. When the material composition was used to modify the dose kernels, calculations improved. The authors suggested that density corrections should be considered for cases in which their PTV includes many slices affected by streaking artifacts [25]. Moreover, beam orientation, device location, and technique of radiotherapy affect the intensity of errors, as a study showed that calculation errors were less intense when an implant in a cylindrical phantom was exposed by VMAT rather than another IMRT plan for both uncorrected and corrected planning CT for metal artifacts [20]. Another study provided mixed results for the role of different MAR algorithms and metal kernels for reducing IMRT dose calculation errors. Interestingly some MAR algorithms increased errors remote from the metal device. The authors showed that unlike calculations in a titanium phantom with simple geometry, metal kernels of CCCS algorithm did not substantially improve dose calculation accuracy in anthropomorphic phantoms of clinical cases (<1% in spine phantom containing titanium rods and dental phantom with amalgam fillings). The authors explained that messy artifacts at metal interfaces had possibly reduced effects of kernels [10]. These data should be taken into account as although there are some surrogate calculations in interfaces but they might not surpass all errors such as contouring and global systematic errors. Knowledge of the geometry and material of device components provided by the implant dentist can be practically implicated by the radiotherapy team and enhance calculations. However, streaking artifacts that are remote from the implant may still affect dose predictions.

This study has some limitations. Firstly, point-to-point comparison is prone to errors since CT scans are reconstructed and contoured films may not replicate their actual volumes. Secondly, only a single lateral field was used to evaluate the effect of implant, that is so simplified for radiotherapy planning. There is less known for the yield of calculation errors on case-by-case basis. It is supposed that complex plans will amend effects of shadow and back scatter dose while the relative effects of each field remain. In future, the effect of high-density materials in adaptive simulations of real cases. (i. e. multiple dental fillings and implants) on dose calculations should be critically evaluated in more sophisticated plans.

## Conclusion

In conclusion, the results demonstrate that high Z materials when included in the radiotherapy field of cancers of oral cavity could cause dose perturbation that interferes with radiotherapy planning. The results of dose measurements of the oral cavity and backscatter suggests that the composition and structural dimension of dental restorations whenever available may be provided for the radiotherapy team and considered in density corrections for oral cancer radiotherapy planning.

## Data Availability

The authors from this work are available to support data.

## Abbreviations

TPS: Treatment planning systems
CCCS: Collapsed cone convolution/superposition
P-F-M: Porcelain fused to metal
ROI: Region of interest
POI: Point of interest
MU: Monitor unit
OD: Optical density
HU: Hounsfield unit
HI: Homogeneity indices
SSD: Source to Surface Distance
UL: Upper layer
DVH: Dose volume histogram
M-Plan: Manually plan
W-Plan: Water plan
D-plan: Default plan
TCP: Tumor control probability

## References

[1] Huang JY, Eklund D, Childress NL, Howell RM, Mirkovic D, Followill DS, et al. Investigation of various energy deposition kernel refinements for the convolution/superposition method. Med Phys. 2013;40(12). 10.1118/1.4831758. 10.1118/1.4831758 PMC3856653 24320507

[2] Maltagliati A, Angiero F, Zaky S, Blasi S, Ottonello A (2018). Reduction of bacterial proliferation by zirconium collar in dental implants. ARRB..

[3] Smeets R, Schöllchen M, Gauer T, Aarabi G, Assaf AT, Rendenbach C, et al. Artefacts in multimodal imaging of titanium, zirconium and binary titanium–zirconium alloy dental implants: an in vitro study. Dentomaxillofac Radiol. 2017;46(2):20160267. 10.1259/dmfr.20160267. 10.1259/dmfr.20160267 PMC5595012 27910719

[4] De Conto C, Gschwind R, Martin E, Makovicka L (2014). Study of dental prostheses influence in radiation therapy. Physica Medica.

[5] Grehn M, Stille M, Ziemann C, Cremers F, Rades D, Buzug TM (2019). A new phantom for individual verification of the dose distribution in precision radiotherapy for head-and-neck cancer. Anticancer Res.

[6] Reft C, Alecu R, Das IJ, Gerbi BJ, Keall P, Lief E, et al. Dosimetric considerations for patients with HIP prostheses undergoing pelvic irradiation. Report of the AAPM Radiation Therapy Committee Task Group 63. Med Phys. 2003;30(6). 10.1118/1.1565113. 10.1118/1.1565113 12852541

[7] Mullins JP, Grams MP, Herman MG, Brinkmann DH, Antolak JA. Treatment planning for metals using an extended CT number scale. JACMP. 2016;17(6). 10.1120/jacmp.v17i6.6153. 10.1120/jacmp.v17i6.6153 PMC5690522 27929492

[8] Hansen CR, Christiansen RL, Lorenzen EL, Bertelsen AS, Asmussen JT, Gyldenkerne N, et al. Contouring and dose calculation in head and neck cancer radiotherapy after reduction of metal artifacts in CT images. Acta Oncol. 2017;56(6):874–8. 10.1080/0284186X.2017.1287427. 10.1080/0284186X.2017.1287427 28464749

[9] Kim Y, Tomé WA (2007). On the radiobiological impact of metal artifacts in head-and-neck IMRT in terms of tumor control probability (TCP) and normal tissue complication probability (NTCP). Med Biol Eng Comput.

[10] Huang JY, Followill DS, Howell RM, Liu X, Mirkovic D, Stingo FC, et al. Approaches to reducing photon dose calculation errors near metal implants. Med Phys. 2016;43(9):5117–30. 10.1118/1.4960632. 10.1118/1.4960632 PMC4991994 27587042

[11] Bouchard H, Lacroix F, Beaudoin G, Carrier JF, Kawrakow I (2009). On the characterization and uncertainty analysis of radiochromic film dosimetry. Med Phys.

[12] Jabbari K, Senobari S, Roayaei M, Rostampour M (2015). Designing and dosimetry of a shield for photon fields of radiation therapy in oral cavity cancer. J Med Signals Sens.

[13] Rayplan 7 reference manual, RSL-D-RP-7.0-EN-1.0-2017-12-08, Sweden, Raysearch Laboratories 2017, Chapter 3, photon dose calculation, 58-61.

[14] Banaei A (2015). Introducing a novel weighted gamma evaluation method for comparing the dose distributions in radiotherapy. Paramedi Sci Mil Health.

[15] Spirydovich S, Papiez L, Langer M, Sandison G, Thai V (2006). High density dental materials and radiotherapy planning: comparison of the dose predictions using superposition algorithm and fluence map Monte Carlo method with radiochromic film measurements. Radiother Oncol.

[16] Wieslander E, Knöös T (2003). Dose perturbation in the presence of metallic implants: treatment planning system versus Monte Carlo simulations. Phys Med Biol.

[17] Saadatmand P, Amouheidari A, Shanei A, Abedi I (2020). Dose perturbation due to dental amalgam in the head and neck radiotherapy: a phantom study. Med Dosim.

[18] Withers HR (2000). Biological aspects of conformal therapy. Acta Oncol.

[19] Rousselle A, Amelot A, Thariat J, Jacob J, Mercy G, De Marzi L, et al. Metallic implants and CT artefacts in the CTV area: Where are we in 2020? Cancer/Radiothérapie. 2020;24(6-7):658–66. 10.1016/j.canrad.2020.06.022. 10.1016/j.canrad.2020.06.022 32859465

[20] Maerz M, Koelbl O, Dobler B (2015). Influence of metallic dental implants and metal artefacts on dose calculation accuracy. Strahlenther Onkol.

[21] Bazalova M, Beaulieu L, Palefsky S, Verhaegen F (2007). Correction of CT artifacts and its influence on Monte Carlo dose calculations. Med Phys.

[22] O'Connor JE (1984). The density scaling theorem applied to lateral electronic equilibrium. Med Phys.

[23] Çatli S (2015). High-density dental implants and radiotherapy planning: evaluation of effects on dose distribution using pencil beam convolution algorithm and Monte Carlo method. JACMP.

[24] Kry SF, Feygelman V, Balter P, Knöös T, Charlie Ma CM, Snyder M, et al. AAPM Task Group 329: Reference dose specification for dose calculations: dose-to-water or dose-to-muscle? Med Phys. 2020;47(3):e52–64. 10.1002/mp.13995. 10.1002/mp.13995 31883390

[25] Parenica HM, Ford JR, Mavroidis P, Li Y, Papanikolaou N, Stathakis S (2019). Treatment planning dose accuracy improvement in the presence of dental implants. Med Dosim.

[26] Lovelock D, Lim S, Yorke E, Kirov A, LoSasso T. SU-E-T-532: Comparison of dose distributions calculated using different planning systems with radiochromic film measurements in an inhomogeneous phantom. Med Phys. 2012;39:3828–8. 10.1118/1.4735621. 10.1118/1.4735621 28518481

